# Amygdala: Neuroanatomical and Morphophysiological Features in Terms of Neurological and Neurodegenerative Diseases

**DOI:** 10.3390/brainsci10080502

**Published:** 2020-07-31

**Authors:** Vladimir N. Nikolenko, Marine V. Oganesyan, Negoriya A. Rizaeva, Valentina A. Kudryashova, Arina T. Nikitina, Maria P. Pavliv, Marina A. Shchedrina, Dmitry B. Giller, Kirill V. Bulygin, Mikhail Y. Sinelnikov

**Affiliations:** 1 Department of Human Anatomy, Sechenov University, 119991 Moscow, Russia; vn.nikolenko@yandex.ru (V.N.N.); marine-oganesyan@mail.ru (M.V.O.); rizaevan@yandex.ru (N.A.R.); vukolova_67@mail.ru (V.A.K.); giller-thorax@mail.ru (D.B.G.); kirill-bh-red@yandex.ru (K.V.B.); 2 Department of Human Anatomy, Moscow State University, 119991 Moscow, Russia; 3 International School “Medicine of Future”, Sechenov University, 119991 Moscow, Russia; arinia2000@yandex.ru (A.T.N.); mariia.pavliv@gmail.com (M.P.P.); 4 Institute for Regenerative Medicine, Sechenov University, 119991 Moscow, Russia; schcedrina-m@mail.ru

**Keywords:** amygdala, limbic system, neurodegenerative disease, pathophysiology of neurodegeneration

## Abstract

The amygdala is one of the most discussed structures of the brain. Correlations between its level of activity, size, biochemical organization, and various pathologies are the subject of many studies, and can serve as a marker of existing or future disease. It is hypothesized that the amygdala is not just a structural unit, but includes many other regions in the brain. In this review, we present the updated neuroanatomical and physiological aspects of the amygdala, discussing its involvement in neurodegenerative and neurological diseases. The amygdala plays an important role in the processing of input signals and behavioral synthesis. Lesions in the amygdala have been shown to cause neurological disfunction of ranging severity. Abnormality in the amygdala leads to conditions such as depression, anxiety, autism, and also promotes biochemical and physiological imbalance. The amygdala collects pathological proteins, and this fact can be considered to play a big role in the progression and diagnosis of many degenerative diseases, such as Alzheimer’s disease, chronic traumatic encephalopathy, Lewy body diseases, and hippocampal sclerosis. The amygdala has shown to play a crucial role as a central communication system in the brain, therefore understanding its neuroanatomical and physiological features can open a channel for targeted therapy of neurodegenerative diseases.

## 1. Introduction

The amygdala was first described in 1826 by Karl Burdach. At that time, nobody knew its exact anatomical structure. Burdach isolated an area currently known as the basolateral nucleus, and named it the amygdala because of its peculiar appearance, resembling the amygdala (almond) nut. With increased interest regarding this new structure, many researchers provided input into outlining the amygdala system, with all its nuclei and connections. Several theories attempted to explain the evolutionary and hierarchy aspects of this region. One theory hypothesized that the amygdala consists of an evolutionary primitive and more recent divisions connected with the olfactory system (the cortico-medial region) and neocortex (the basolateral region) respectively [1]. This complex receives information from sensory analyzers, assigns it a specific value and influences the behavioral response. This process plays a role in emotional responses such as an anger, fear, and aggression. Emotions are a subjective perception of an individual’s internal state, particular needs, and numerous outside influences, such as social and environmental factors. The brightest emotional sensations are associated with human interaction. In the nineteenth century, it was found that animals and humans have similar responses to different triggers. Since then, significant research has been performed in order to better understand brain activity.

Previously, it was thought that the hypothalamus was connected with only the cortex and brainstem, but in 1937, the temporal structures were shown to be involved in emotional expression. The limbic system was introduced in 1878 by Broca on a comparative anatomical basis. McLean connected the limbic system to include the amygdala. Lesions in the amygdala were known to lead to “psychic blindness”. This showed that this structure is one of the most important emotional response centers. In recent studies, it was discovered that the amygdala can accumulate misfolded pathological proteins throughout life, which is hypothesized to play an important role in neurodegenerative disease etiology.

## 2. Materials and Methods

**Figure 1:**
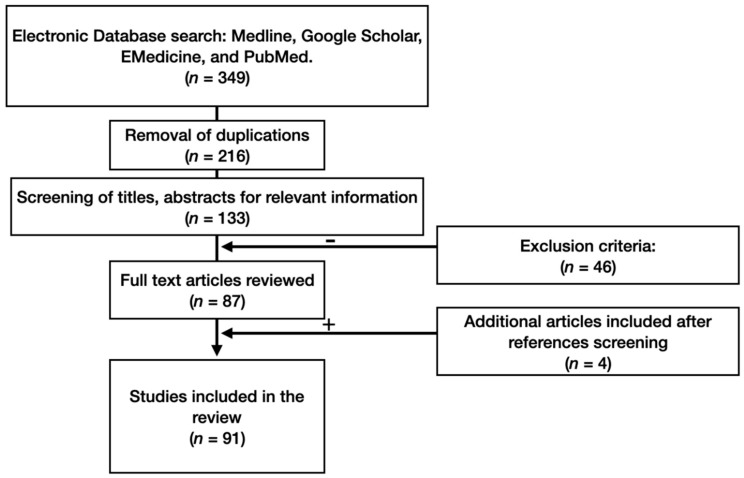
Flow-chart of study methodology.

A thorough literature search for information on neuroanatomical and morphophysiological features of the amygdala in terms of neurological and neurodegenerative diseases was carried out, using the databases of Medline, Google Scholar, EMedicine, and PubMed. We studied the references and conducted a citation search. The PICO model formed the basis of the search strategy. Foreign language material was included in this study. The various mechanisms for searching in our study generated a total of 87 publications. Four additional publications were identified as the study progressed (Figure 1).

Primary evaluation of the articles indicated that our search strategy had identified a large number of irrelevant studies. Criteria to eliminate studies that did not address our research question were developed throughout articles processing. Primarily, we included original research articles and case studies that examined the amygdala in terms of neurodegenerative diseases. We later included anatomical studies examining amygdala neuroanatomy, communication and physiology. We excluded case reports and non-conventional studies. These criteria identified 213 articles for review. Next, we narrowed our inclusion criteria to articles published after 2000, with the exception of articles of historical value, which identified a total of 91 articles. Two co-authors independently selected, evaluated, and extracted data. Keywords of the strategy of the search were the combination of the following terms: amygdala, anatomy, neurophysiology, neurodegenerative, limbic system, morphology, pathology, neuroanatomy.

## 3. Results

Over 1200 papers were primarily found according to search criteria. After thorough selection in regard to date of publication, context and availability, 91 papers were selected as suitable to write an update on the neuroanatomical and morphophysiological features of the amygdala in regard to neurodegenerative disease and neuroanatomy. A general consensus in the reviewed literature shows that the anatomical features of the amygdala as well as biochemical and physiological markers within the complex amygdala system play a crucial role in the pathological mechanisms of neurodegenerative diseases. In order to better understand the integrative role of the amygdala in brain function and communication, we chose to discuss the anatomical aspects of the amygdala and its role in neurological and neurodegenerative diseases.

### 3.1. Anatomy of the Amygdala

The amygdala is part of the limbic system, averages 1.1–1.7 cm^3^ in volume, and is a crucial structure in the brain, performing three main functions: expression of emotions, memory processing and managing stimulatory input [1]. The limbic system mainly consists of the thalamus, hypothalamus, hippocampus, amygdaloid complex of nuclei, basal ganglia and cingulate gyrus. It is located deep within the brain, above the brainstem and underneath the cerebral cortex [2]. The amygdala as defined as located in the most ventrocaudal part of the brain, near the hippocampus, in the frontal portion of the temporal lobe, below the subcortical nuclei, expanding to its basal structures. It borders with the secondary olfactory areas and paleocortex structures. The fibers of the visual tract cross medially from the amygdala. Caudally, it borders with the structures of the hippocampal formation. Laterally, the amygdala nuclei are covered by the piriform cortex, and in the most caudal parts—by the entorhinal region [1,2].

Using cytoarchitectonics studies and neural organization of the amygdaloid complex, an evolutionary–morphological classification of the amygdala structures has been proposed [3]. The central core, insertion masses, lateral and basolateral cores have the character of typical nuclei, i.e., they are compact, well-bounded by fibrous layers, and have similar morphology. However, they possess cytoarchitectonic features in a structural organization similar to other cell populations (the nucleus of the lateral olfactory tract, the periamygdaloid and piriform cortex, the lateral part of the posterior cortical nucleus). On this basis, they represent formations of the paleocortex. The amygdaloid cortex also includes many clusters of neurons which cannot be attributed to either nuclear or paleocortical formations. These clusters are located on the basal surface of the brain, on the plexiform layer, which contains afferent axons coming from the olfactory bulbs. These neurons possess polymorphism, which is manifested both by the size and shape of the neurons, and their cytochemical properties. In different zones of these clusters, different densities of neurons are determined, but there is no separation into layers with the formation of plexuses. Such clusters of neurons have the character of transient, intermediate, or simply intertidal formations located in different regions of the core. They are also described as intermediate between the nuclei and the paleocortex [3].

The amygdala includes six main nuclei: the central, cortical, medial, accessory, basal and lateral nuclei. They can be divided into three groups depending on their location: the deep or basolateral, the superficial or cortical-like group and the centro-medial group [4]. The basolateral nuclei consist of the lateral (which is the largest in human brain and usually asymmetric) basal and accessory nuclei, and each of them are subdivided into other nuclei (Table 1). These nuclei receive signals from the cortex, thalamus and hippocampus [1,4,5].

The amygdala can be divided into the superficial region and transition zone with the entorhinal cortex. The cortical nucleus is a superficially layered structure at the surface of the brain and consists of the anterior and posterior cortical nuclei, the lateral olfactory tract, the bed nucleus of the accessory olfactory tract, the periamygdaloid cortex. This area is shown to be the one of the first affected by neurodegenerative diseases. Cortical nuclei are connected with the olfactory bulb and hippocampus. This area manages our memory storing, the processes of thinking, feeling and sensing [1,4].

The centromedial nucleus is formed by the central (CeA) and medial (amygdaloid part of the bed nucleus of the stria terminalis) nuclei, and is located in the dorsomedial part of the amygdala (Table 2).

The amygdala receives blood supply from the internal carotid and basilar arteries, especially from the anterior and posterior cerebral arteries, anterior choroidal artery. Pathologies in such a widespread vascularization system provides an opportunity for the development of lesions in the amygdala [4].

### 3.2. Neuronal Complexes of the Amygdala

#### 3.2.1. Basolateral Neural Complex

There are several distinguished types of cells found in the basolateral neural complex in regard to morphology. The first type is called type I or pyramidal-like and these cells have spiny basal and apical dendrites with equal length, a pyramidal-like form, organized randomly without an exact location in one plane [6,7,8,9,10]. The axons are derived from the soma and spot, where the primary dendrite appear. They are similar morphologically to neurons of the basal nucleus of the amygdala (BA) and lateral nucleus of the amygdala (LA) with a larger diameter of the soma in the LA [7,9]. Dendrites of the pyramidal neurons are located on the verge between different subdivisions. Therefore, it is suggested that this can play a role in the functional differentiation between subdivisions of the nuclei [8,11].

Another type of cells, especially GABAergic (II type or stellate) are smaller and without spines on the dendrites [12,13]. It is suggested that there are several types of interneurons based on their chemical expressions [14,15,16]. Authors of several studies distinguished several other types of cells based on their axonal and dendrite features: cone, chandelier, extended and neurogliaform neurons [6,7,9,17,18].

Physiologically, the neurons of the LA are divided into three groups depending on their action potential: the first type (pyramidal or projection neurons) have broad action potential (95%) and the second (interneurons) have a short action potential [11,19,20]. The third type (3%) is distinguished as a discrete type because of their single-fire activity [8,17,21,22]. The firing patterns of these neurons are determined by calcium-activated potassium channels and a variety of voltage-gated channels [8,23]. There are two groups of cells in the BA distinguished by their electrophysiological features: pyramidal cells (95%), which are subdivided into repetitive (late-firing) and burst firing (producing one or two spikes due to current injections) neurons, and a second group of interneurons (5%) [22,24,25].

The synaptic features of the basolateral neural complex of the amygdala (BLA) are also uniquely specific. There are three types of ionotropic glutamate receptors within the BLA: the AMPA (Alpha-amino-3-hydroxy-5-methyl-4- isoxazolepropionic acid), NMDA (N-methyl-D-aspartate acid) and Kainate receptors, and one type of metabotropic receptors [26]. The inputs, which go to the BLA from the cortex and thalamus, are transferred to electrochemical signals within synapses containing AMPA and NMDA receptors [27,28]. The products of four genes (*GluR1–4*) unite to form the AMPA receptor. Calcium penetrability depends on its composition: the absence of *GluR2* leads to a high level of calcium permeability [29,30]. The NMDA receptor consists of four to five parts: two NR1 and two–three NR2 subdivisions. It has been shown that NR2 subunits contain NR2A or NR2B, and the latter changes to NR2A during development [31,32]. Often, this receptor is deactivated using extracellular Mg^2+^ [33,34]. Thalamic inputs have a smaller amount of these blocked receptors which is why they are excited at resting potential [35,36]. There are groups of intercalated neurons in the BLA. Different cells can receive stimulating inputs from the core and thalamus, and mediate inhibition in both directions [13,19,37,38].

#### 3.2.2. The Central Nuclei

Morphologically, there are several types of neurons located in the central nucleus of the amygdala (CeA). In the lateral sector of the central nucleus, a predominant cell type with ovoid soma is located. These cells have several primary nonspiny dendrites, branching onto spiny secondary and tertiary dendrite. Their axons begin branching even before leaving the nucleus, which is why these cells are called “medium spiny neurons” [12,39]. Another type of neurons located in the central nuclei have big soma with thick aspiny dendrites, branching on to secondary seldom spiny processes [39,40,41]. The third type of cells are small aspiny neurons [40]. These cells spread uniformly in the CeA. It has been shown that there are different peptides, dividing cells into two groups: the first group has enkephalin, the second group—corticotropin releasing hormone [42]. It is suggested that such neurons with different chemical content have different functions [43,44].

In regard to physiological aspects, there are few studies about electrophysiological properties of the neurons of the CeA. Two distinct cell types were described in terms of their reaction to a prolonged and short current injection: type A (75%) and type B (25%) [41,45]. Another two types of cells called “burst-firing” cells and “fast-spiking” cells are present in the amygdala. Most of the internuncial neurons are fast-spiking cells showing no adaptation and high frequency of rapid action potential [46]. Burst-firing cells can be subdivided into two groups: group I has adaptation and shows recommencing bursts of action potentials (making them alike to the B-type cells described above), and group II has neurons with low-threshold burst activity, which are similar to type A cells, described above [45,46]. However, the discrepancies between these two groups can be explained due to the differences in research techniques. For example, the number of late-firing neurons is different depending on experimental protocol [47].

Cells in the central nuclei lateral division (CeL) and central nuclei medial division (CeM) have different morphological and electrophysiological activity, but there is no correlation between these two criteria [45]. According to several studies, the glutamatergic signals from the lateral and basal nuclei go to the neurons of the CeL and CeM and activate AMPA and NMDA receptors [48,49]. These inputs trigger an activation of presynaptic metabotropic glutamate receptor expression, which inhibits synaptic input. In epilepsy, the changes in synaptic activation play an important role [26,50,51,52].

Synaptic features of CeA are well discussed in literature. There are two types of GABA ionotropic receptors in the lateral group neurons of the central nucleus. The first type can be inhibited by bicuculline, 1,2,5,6-tetrahydropyridine-(4-yl)methylphosphinic acid (TPMPA) [53], and activated by barbiturates and benzodiazepines [54]. Due to their chemical similarity to GABA_C_ receptors in the retina, they are called GABA_C_-like receptors [55,56]. They spread widely in the central nucleus and in the medial subdivision [49]. The second type presents in the CeL nucleus. The interneurons form synapses with dendrites of CeL nuclei which express synapses with both types of receptors. Impulses from the dorsomedial region interact with the soma of the central nucleus neurons, which have GABA_A_ receptors [57]. The beginning of CeL neuron axons is spiny [39]. It is suggested that if the synapses occur between the soma and these spines, then their excitation can be inhibited. Different GABA-like receptors maintain different types of track signals in the amygdala. [57] The amygdala is sensitive to benzodiazepine, therefore this chemical can affect this brain structure and lead to its inhibition via impact on the GABA_A_ receptor [58,59]. This fact can explain benzodiazepine’s anxiolytic effect on the organism.

### 3.3. Intercalated Cell Masses

The interneurons with GABA-like receptors are directly connected with cells of the CeA. This link leads to the formation of an inhibitory signal [49,57,60,61]. There are two types of interneurons, distinguishing by their size, form, dendrite branching, localization in regard to the boundaries with other nuclei [61,62]. These cells are characterized by an enhanced irritability because of the prolonged afterdepolarization and they play a crucial role in regulation of the CeA [63]. Additionally, these neurons inhibit the activity of the medial neurons because of their orientation from the lateral border to the medial [64]. Such an organization leads to specific control over the central nucleus’s activity [64].

The medial nucleus consists of one cell type similar to the CeM neurons [65]. These neurons receive olfactory signals (NDD) as long as visceral, sensory, and autonomic input. This is often referred to as the extended amygdala, which includes the bed nucleus of the stria terminalis and sublenticular region, the cetromedial nucleus.

### 3.4. Input and Output Connections

Mostly through animal studies using retrograde and anterograde injected tracers into different cerebral areas, it was found that the amygdala has widespread connections with other brain structures [66,67,68]. Input signals come to certain nuclei of the amygdala, then the information is processed and output signals are generated to other cortical and subcortical regions.

The sensory and memory information afferent signals from the cortex and thalamus, and signals from the vegetative nervous system ascend from the brain stem and hypothalamus to the amygdala [66]. Most cortical inputs pass through the external capsule [69]. These signals can be mono- or polymodal.

**Figure 2:**
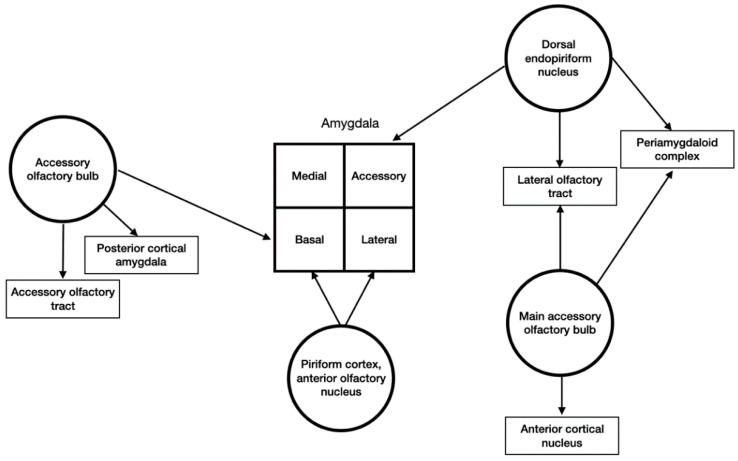
Schematic representation of olfactory sensory input to the amygdala.

All of the olfactory associated regions have connections with the amygdala (Figure 2). Olfactory signals arise from the primary olfactory cortex, endopiriform nucleus, the accessory and main olfactory bulbs [4].

**Figure 3:**
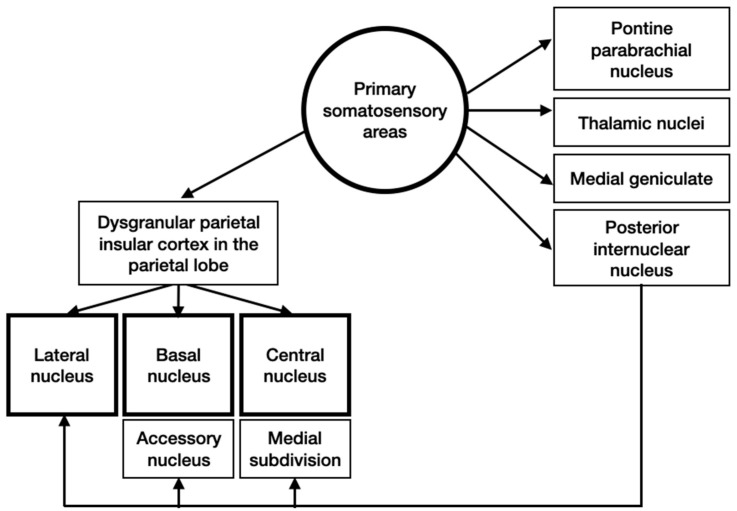
Schematic representation of somatosensory inputs to the amygdala.

Inputs from primary somatosensory areas reach the amygdala primarily from the dysgranular parietal insular cortex in the parietal lobe (Figure 3). Then, these signals pass through different structures and come to different nuclei of the amygdala.

**Figure 4:**
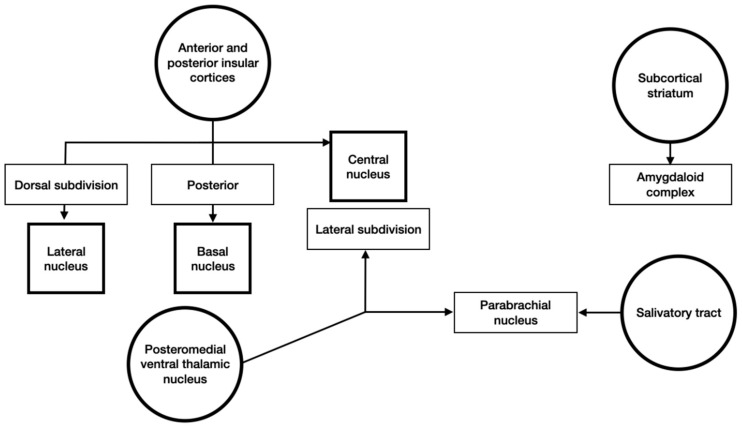
Schematic representation of gustatory and visceral inputs to the amygdala.

Gustatory and visceral information arrives from the anterior and posterior insular cortices and the subcortical structures (Figure 4). This was cortical and subcortical signals focus in the amygdaloid complex.

Auditory and visual information is sent to the amygdala from associated areas and rarely from the primary cortex. Signals from subcortical areas via the thalamic medial geniculate nucleus reaches lateral nucleus of the amygdala. 

**Figure 5:**
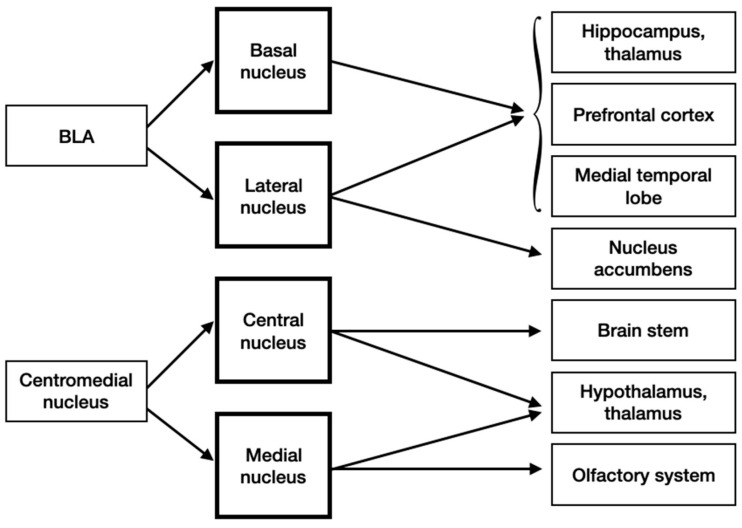
Efferent inputs into the amygdala.

The hippocampus, thalamus, prefrontal cortex and medial temporal lobe get projections from both the BA and LA. Additionally, the nucleus accumbens receive information from the LA. The brain stem, which is involved in autonomic response to fear, and hypothalamus receive input from the central nucleus of the centromedial nucleus, the last two and the olfactory system obtain signals from the medial nucleus (Figure 5).

**Figure 6:**
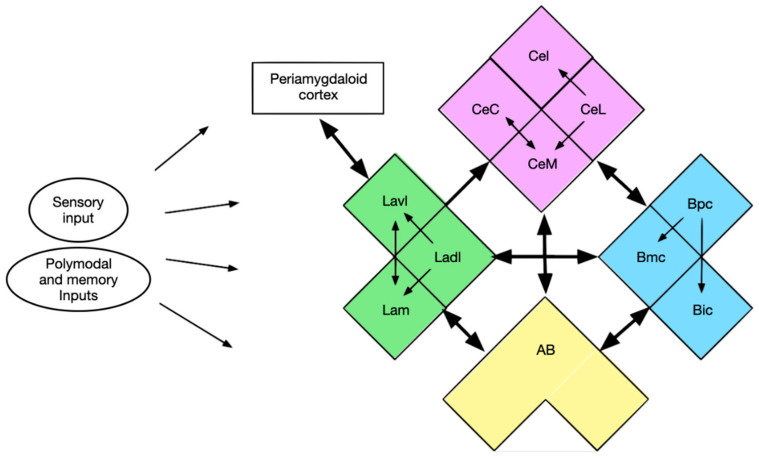
Intra-amygdaloid links (Green—LA: Lavl–ventrolateral subdivision, Ladl—dorsolateral subdivision, Lam—medial subdivision; Purple—central nucleus: CeC—capsular subdivision, CeM—medial subdivision, CeL—lateral subdivision, CeI—intermediate subdivision; Blue—basal nucleus: Bpc—parvicellular subdivision, Bmc—magnocellular subdivision, Bic—intermediate subdivision; Yellow—accessory basal nucleus—AB).

A vast variety of input signals reach the amygdala and its numerous nuclei. However, there is also a widespread network interconnecting the nuclei and their subdivisions (Figure 6). All nuclei connect with each other and also have reciprocal links except the LA and CA. In some nuclei (LA and BA) there are rostrocaudal connections between subdivisions.

### 3.5. Neurodegenerative Diseases: Hypothesis of Pathologic Synergy in the Amygdala

**Figure 7:**
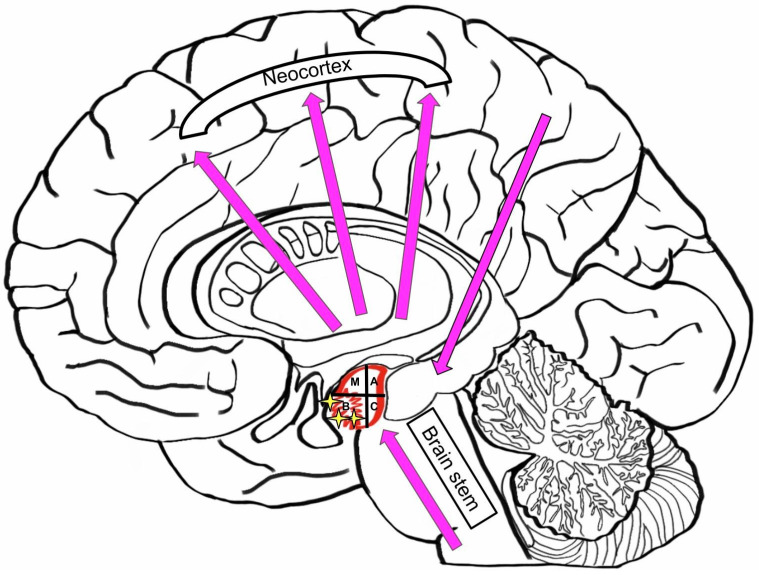
Dysfunctions in the brain effected by Alzheimer’s Disease (M—medial nucleus; A—Accessory nucleus; B—basal nucleus; C—caudal, or lateral nucleus).

Neurodegenerative diseases are often associated with age [70]. Throughout life, the human brain accumulates different proteinaceous aggregates, such as Tau and Aβ. This pathological storing has a synergetic mechanism between Tau and Aβ, and can lead to primary age-related tauopathy (PART) and Alzheimer’s disease (AD) [71,72]. Different misfolding proteins (Tau, Aβ, α-Synuclein, and TDP-43) can be found in the amygdala [1]. Different brain structures, such as neocortical, allocortical and brainstem structures, are directly connected to the amygdala, and are therefore affected by the disfunction of each other. Atrophy in the basal nuclei, accumulating Aβ plaques in the cortical transition zone, and loss of cells in the corticomedial group may lead to AD (Figure 7). Late AD is associated with large amounts of Aβ amyloid and Tau aggregates in the amygdala. The appearance of TDP-43 and a-SN testifies to an advanced AD. AD is directly associated with genetic mutations in *PSEN1* and *PSEN2*, which lead to the accumulation of a-SN pathology in amygdala and AD [5].

**Figure 8:**
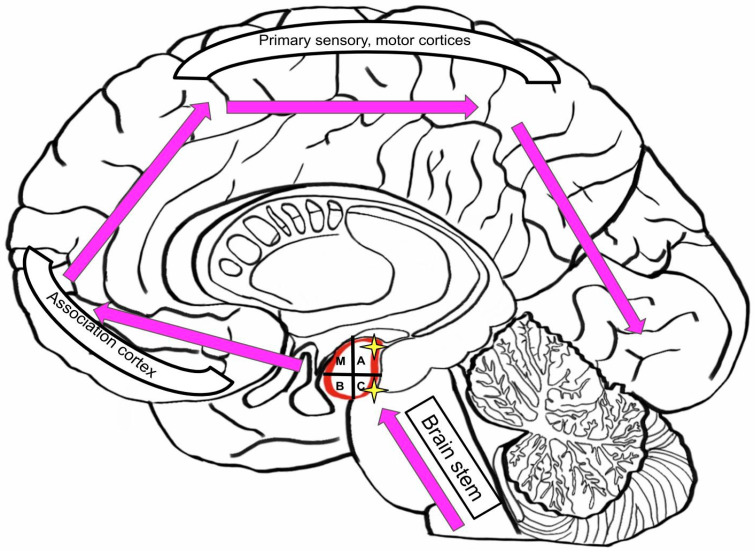
Similar dysfunctions patterns in the brain effected by Lewy Body Disease (LBD) and Parkinson’s Disease (PD).

Lewy Body Diseases (LBDs), such as dementia with Lewy bodies and Parkinson disease (PD), develop in a certain pattern from caudal to rostral: spreading from brain stem to midbrain and limbic lobe, then reaching neocortex and finally affecting the primary, sensory and motor cortices [73]. The accessory and central nuclei are primarily affected in the amygdala (Figure 8).

TDP-43 pathology has massive tropism to the amygdala, and nowhere else. Additionally, this pathology is synergetic with neurofibrillary triangles (NFTs) or Tau diseases. It is important to research new therapeutic and diagnostic targets in the amygdala because of its potential benefits in the treatment of AD and other neurodegenerative diseases. This is mainly due to the important neuroanatomical and neurophysiological aspects of the amygdala, as a central processing unit in the brain.

Depression is common in AD patients, with radically declining quality of life [74,75]. Unfortunately, there is no beneficial pharmacotherapy for such cases, because monoaminergic antidepressants have not shown efficacy [76,77]. It was reported using neuroimaging that these patients have changes in the grey and white matter, and other important brain structures: the prefrontal and temporal cortex, corpus callosum, the posterior cingulate gyrus [78,79,80,81,82]. Dekens et al. reported that in patients with AD depression, the amygdala is involved. The specific markers of this case are receptors of the neutrophil gelatinase-associated lipocalin protein [83]. Patients with both AD and depression have increased functional connectivity between the orbitofrontal and medial prefrontal cortex with the left amygdala. This could be a main reason for AD associated depression. Similar findings were noted in patients with dementia, showing that apathy was prevalent in patients with neuroanatomical disfunction of central brain structures, including the amygdala [84].

Zhongwei Guo et al. suggested that a special role in the disease belongs to an increased inhibition of the amygdala by the orbitofrontal cortex. In this case, acupuncture therapy is prescribed to regulate the cooperation between the medial prefrontal cortex and the amygdala. A lesion in the amygdala, decreasing functional connection between it and inferior frontal gyrus, has been diagnosed in patients with depression and Parkinson’s disease [85]. Furthermore, a decreased right functional connection between inferior frontal gyrus, medial prefrontal cortex and the amygdala plays a prominent role in the development of depression in patients with AD.

Three types of neurons are involved in the process of controlling the activity of different brain neurons: neuroglial cells, GABAergic interneurons and the glutamatergic pyramidal. The amygdala has all of these regulatory cells, playing a crucial role in behavior regulation. The stimuli from stria terminalis and LA primary go to the BLA. Furthermore, the outputs play a significant role in the functioning of the nucleus accumbens, prefrontal cortex and hindbrain [86].

### 3.6. Role of Amygdala in Neurological and Neurodegenerative Diseases

Through an understanding of the intricate mechanisms of neurophysiological and neuroanatomical aspects that place the amygdala as a central regulatory system, it is hypothesized that amygdala pathology can have a chaotic effect on brain regulation, resulting in conditions ranging in severity and impact.

In cases of drug addiction, the BLA neuron dysfunction and exactable outputs to nucleus accumbens are observed [86]. BLA plays a role in central reinforcement mechanisms. Lesions in this region can cause failures in the reward assessment system, as shown in experiments with opiates. In the case of early opiate exemption, the value of water and sucrose rewards increases [86]. Withdrawal negative effects are predominantly induced by the amygdala [87].

Post-traumatic stress disorder (PTSD) is intricately associated with certain amygdala misfunction. Risks of development of generalized anxiety and PTSD increase because of traumatic occasions and have shown to inhibit the work of GABA_A_-receptors in the α_2_-subunit of the amygdala [85].

The amygdala has a prominent role in memory formation. In dangerous situations, corticosteroids and other stress hormones are released. This enhances memory consolidation within the amygdala [87]. Damage in the BLA disturb these hormonal effect and recognition functions. Different experiments have established that if the amygdala has lesions, and infusions of glutamate into the hippocamp, the activation of cAMP cascades in the cortex of hippocampal formation do not work as facilitating manipulations for memory formation [87]. However, memory consolidation can also appear without activity in the amygdala. This was proved by many experiments with mazes and lidocaine injections in the amygdala [88,89]. Refusal of opioids has a positive effect on memory formation, as patients taking methadone showed an increased memory function after spontaneous withdrawal [90].

The amygdala plays a main role in the circuits of fear because most of the information about fear comes to the amygdala and stimulates it. This structure mediates fear learning [91,92]. Fear forms memory due to cooperation and interaction between conditioned and unconditioned stimuli, and later it can appear under the influence of conditioned stimulus. The mechanisms involved in fear can help us better understand different anxiety disorders [93]. In human amygdala, the brain-derived neurotrophic factor is essential for forming memory of fear. Polymorphism in the gene of this factor can cause different perceptions and analyses of fear like stress-induced anxiety. However, McGaugh et al. reported that lesions in the amygdala do not depress faculty of fear retention [86,94]. With its essential role as an emotional regulatory system, the amygdala has shown to be central to emotional processing dysfunction in patients with bipolar disorder, anxiety, depression [95].

Norepinephrine stress-activated β-adrenergic receptors, located on the BLA principal neurons, alter afterhyperpolarization, decreasing them. Because of this, neuronal excitability increases [86]. Basolateral amygdala forms memory traces because it receives and stores conditioned (CS) and unconditioned stimuli (US), like food reward or shock [94]. CS obtains an ability to make different responses when it is associated with US, and the answer is either defense or reward. Different behavioral strategies appear in the amygdala, especially in the BLA and LA, because specific CS–US connections are formed within them [93]. 

The amygdala is engaged in the formation of fear and anxiety behavior. The blockage of BLA leads to the cessation of anxiety responses. PTSD has been shown to be associated with amygdala disfunction, and potential targeted treatments have been introduced [96]. Experiments describing the formation of anxiety in rodents were conducted with combinations of CS—auditory and US, presented as R—reward, N—neutral, S—shock. After making a CS–R pair, a rat developed a food-seeking behavior, and after CS–N—nothing happened, but when a shock was presented, rodents froze even if this dangerous US stopped affecting them. It was shown that in the process, different behavior groups of neurons were engaged, their activity increased under one CS impact [97], they had no overlap and were inhibited or stayed unresponsive. When CS–S affected them, a small number of neurons became activated, which leads to freezing. With the functional MRI (fMRI), the suggestion that the anxiety is associated with changes in the resting activity of these neurons was proved. It is interesting to note that Pankaj Sah, in his work [98], found that while learning CSs, more neurons are inhibited compared to exited ones. Thus, the activity of a small group of neurons in the BLA provides the neural substrate with different psychiatric disorders [98]. Still, it is not understood how this activity appears and sustains. It is important to investigate this subject because a more specific target therapy for these disorders can be found through impacting on BLA neurons [98]. Benzodiazepines have been shown to have an anxiolytic effect on the organism, due to their inhibitory impact on the GABA_A_ receptor in the amygdala [58,59].

The amygdala plays an important role in regulating human emotions. It is provided by its connections with the medial prefrontal and the orbitofrontal cortex, inferior frontal gyrus, hypothalamus, and the ventromedial striatum [99]. In patients with major depressive disorder, lesions are found to impact the transfer of information from the orbitofrontal or prefrontal cortex and the amygdala, with a decrease in their activity [100,101].

In neurodegenerative diseases, the amygdala has been shown to play an important role in the disassociation of brain system communication and behavioral output. Neurodegeneration is seen in postmortem brain dissection in patients with Alzheimer’s disease [102]. Studies have shown that in Alzheimer’s disease, as well as other neurodegenerative diseases (Pick disease, argyrophilic grain disease, Lewy body dementia), Lewy bodies are often abundant in the amygdala complex [103,104]. Pathologically misfolded proteins, common in many neurodegenerative diseases, are often found to be concentrated in the amygdala, specifically, it has been shown to be a locus of pathologic synergy Tau, Aβ, α-Synuclein, and TDP-43 misfolding with tropism for the amygdala [5,105]. The abundance of knowledge on the accumulation of misfolded proteins shows high variability between each clinical case, with some individuals having high tropism to the amygdala, while others have minimal accumulation. Underlying conditions in individual brains may favor the misfolding of multiple proteins, yet the most acceptable notion to this date is that a cooperation of influencing and predisposing factors, including environmental, phenotype-dependent and genetic are responsible for target-specific protein accumulation. Amygdala atrophy has been shown to be prominent in early stages of Alzheimer’s disease, with correlation between the extent of atrophy and disease severity [106,107]. These findings expand on early studies, hypothesizing the amygdala as a central participant in Alzheimer’s disease pathology [108].

As a central information processing organ, the amygdala integrates exteroceptive information to the isocortex, limbic system and endocrine and autonomic regulatory centers. In Parkinson’s disease, specific lesions arise in the amygdala, contributing to behavioral changes and autonomic dysfunctions [109]. Parkinson’s patients show decreased functional connectivity between the amygdala and fronto-parietal areas, more severe with depressed patients. The severity of amygdala disfunction is associated with depression severity in patients with Parkinson’s disease [110]. Furthermore, signs of amygdala hypotrophy are more often seen in Parkinson’s disease patients with anxiety symptoms [111]. 

These findings suggest the undoubtable role of the amygdala complex in disease progression, with the theoretical possibility of new targeted treatment protocols. The anatomical characteristics of the amygdala complex indicate widespread communication of the amygdala within the brain, acting as a central processing unit. Due to lack of knowledge on complex communication mechanisms within the brain, the complete function of the amygdala remains unknown. Despite this, current literature suggests that the amygdala may play an important role in many known neurological and neurodegenerative diseases, acting as a central organ of misinterpretation of information, leading to specific clinical manifestations. Finding specific targets for therapy with focus on the amygdala could prove to be beneficial in solving many outstanding clinical problems.

## 4. Discussion

Recent discoveries allow us to better view the importance of the amygdala, as one of the most central brain structures in the limbic system, because of its widespread connections with other brain structures. It is involved in the processes of memory formation, fear behavior, anxiety symptoms, encouraging mechanism and many other regulatory functions. The amygdala consists of six main nuclei, of which the accessory and central nucleus are most commonly affected in degenerative diseases [73].

Different misfolding proteins (Tau, Aβ, α-Synuclein, and TDP-43) can be found concentrated in the amygdala [1]. Atrophy in the BA, the storing of Aβ plaques in the cortical transition zone, and the loss of cells in the corticomedial group may lead to AD [73]. This important brain structure has the main role in depression formation in patients with AD, because of increased functional connectivity between the orbitofrontal and medial prefrontal cortex [84]. Lesions in the BLA lead to disorders in the reward assessment system [85]. It is interesting to note that the blockage of BLA leads to termination of anxiety [94]. During stress induced situations, there is an excretion of corticosteroids and other stress hormones, which enhance memory consolidation within the amygdala [86].

All of the discussed neuroanatomical and neurophysiological aspects of the amygdala place it at the center of many known pathological mechanisms of neurodegenerative diseases. As a crucial interconnecting structure in the brain, the amygdala may play a decisive role in degenerative disease progression, through misdirecting brain communication, restricting memory formation, and impacting behavioral mechanisms and important cognitive functions. Undoubtedly, such an important structure in connecting different areas of the human brain can play a central role for targeted disease treatment. Existing evidence, discussed in this review, supports this hypothesis.

## Figures and Tables

**Table 1:** Division and subdivision of basolateral amygdala nuclei according to location.

Nuclei	Location	Subdivision
Lateral	Dorsally in the amygdala, ventrally is the basal nucleus	Dorsolateral, Ventrolateral, Medial
Basal	Ventral to the lateral nucleus	Rostral magnocellular, Caudal intermediate, Parvicellular
Accessory	Ventral to the basal nucleus, near by the amygdala–hippocampus area	Magnocellular, Intermediate, Parvicellular

**Table 2:** Division and subdivision of centromedial amygdala nuclei according to location.

Nucleus	Location	Subdivision
Central	In the rostral part of the amygdala, medially to the basolateral nuclei, laterally to the stria terminalis, ventrally to the globus pallidus.	Capsular, Lateral, Intermediate, Medial
Medial	Laterally to the optic tract	Rostral, Central (Dorsal and Ventral), Caudal
